# 

**Published:** 1861-05

**Authors:** 


### CONTENTS.

### ORIGINAL COMMUNICATIONS.

PAGE.
ARTICLE I.—Annual Address. Delivered before the Medical Association of the State of Georgia, at its Session in Atlanta, April 11th, 1861. By A. Means, M. D., LL. D. .......... 503
ARTICLE II.—Report of the Proceedings of the Medical Association of Georgia, for 1861 .......... 529
ARTICLE III.—The Etiology and Treatment of Typhoid Eever. By V. H. Taliaferro, M. D., of Columbus, Ga., .......... 538
Hip-joint Dislocation Reduced by Manipulation. By Z. P. Landrum, M. D., Columbus, Miss., .......... 551

### SELECTIONS.

Letter from Berlin .......... 557

### EDITORIAL AND MISCELLANEOUS.

American Medical Association, .......... 563
Atlanta Medical College, .......... 564
List of Payments, .......... 564

